# Cumulus cells enhance oocyte genomic quality control by promoting DNA damage-induced meiotic arrest

**DOI:** 10.1242/jcs.264929

**Published:** 2026-07-27

**Authors:** Chenxi Zhou, Brett McKinnon, Hayden Homer

**Affiliations:** ^1^ Christopher Chen Oocyte Biology Research Laboratory, UQ Centre for Clinical Research, Building 71/918, Royal Brisbane & Women's Hospital Campus, Herston, Queensland 4029, Australia; ^2^ Institute for Molecular Bioscience, University of Queensland, St Lucia, Queensland 4067, Australia

**Keywords:** Oocyte maturation, Cumulus cells, DNA damage response, Cumulus–oocyte communication, Genome integrity, Spindle assembly checkpoint

## Abstract

Cumulus cells are known to maintain oocyte arrest at prophase I through gap junction-mediated cAMP signalling, but their role after meiotic resumption remains unclear. Here, we show that cumulus cells enhance oocyte genomic quality control by sensitizing mouse oocytes to DNA damage-induced meiotic arrest. Time-lapse imaging of SiR-tubulin-labelled spindles revealed that oocytes from cumulus–oocyte complexes (COCs) matured faster than denuded oocytes (DOs). Upon mild DNA damage induced by low-dose etoposide, COC oocytes arrested at metaphase I, whereas DOs completed maturation despite similar levels of DNA lesions. This arrest required spindle assembly checkpoint (SAC) activity, as reversine rescued polar body extrusion and BubR1 and Mad2 were elevated in COCs but not DOs. Disruption of gap junctions or inhibition of mTOR signalling abolished the checkpoint response. Notably, cumulus cells did not enhance oocyte response to minor spindle perturbations. These findings reveal a previously unrecognized role of cumulus cells in mediating DNA damage-induced SAC activation, providing post-GVBD genomic surveillance beyond prophase I arrest.

## Teaser

**Highlighted Article:** Surrounding support cells help immature eggs respond to DNA damage by halting cell division, providing an additional quality-control mechanism that may protect fertility and embryo health.

## INTRODUCTION

Oocyte meiotic maturation is a tightly regulated process essential for the generation of a competent gamete capable of fertilization and embryonic development (Li and Albertini, 2013; Conti and Franciosi, 2018). In mammals, oocytes are surrounded by cumulus cells, forming the cumulus–oocyte complex (COC), which provides metabolic support, paracrine signals and coordinated communication through gap junctions (Eppig, 2001; Sugiura et al., 2007; Del Bianco et al., 2024; Marchais et al., 2022). These interactions are well known to maintain oocyte arrest at prophase I through a cGMP–cAMP signalling network, whereby cGMP generated in the surrounding follicular somatic cells is transferred to the oocyte via gap junctions and suppresses PDE3A activity, thereby maintaining elevated oocyte cAMP levels and preventing meiotic resumption (Conti and Franciosi, 2018; Li and Albertini, 2013; Pan and Li, 2019; Pei et al., 2023). This physiological regulatory mechanism ensures that meiotic resumption occurs only in response to appropriate hormonal cues.

Beyond this classical role, the functions of cumulus cells after meiotic resumption remain poorly understood. It is generally assumed that following germinal vesicle breakdown (GVBD), cumulus cells become functionally decoupled from the oocyte and do not significantly influence subsequent maturation events, including spindle assembly, chromosome segregation and polar body extrusion (Albertini et al., 2001). Consequently, many studies on oocyte quality control rely on denuded oocytes (DOs) as a model system, potentially overlooking contributions from the surrounding somatic cells.

It has been well known that SAC plays crucial roles in preventing the progression of spindle damaged oocytes through meiosis (Pun and North, 2025; Mukherjee et al., 2024). However, mammalian oocytes are traditionally considered to possess a relatively weak SAC compared with somatic cells, as reflected by frequent aneuploidy and limited arrest in response to spindle or DNA damage (Homer et al., 2005; Rieder and Maiato, 2004; Sanders and Jones, 2018). This raises the question of whether cumulus cells contribute to oocyte checkpoint responses, particularly under conditions of DNA damage, which are common during *in vitro* maturation and ageing (Ammar et al., 2024; Schumacher et al., 2021; Sun et al., 2024).

Here, we investigate the dual role of cumulus cells in regulating oocyte maturation. First, we examine how cumulus–oocyte interactions influence the timing of meiotic progression using time-lapse imaging. Second, we test whether cumulus cells sensitize oocytes to DNA damage-induced metaphase I arrest and assess the underlying mechanisms. By combining pharmacological inhibition of SAC, gap junctions, and mechanistic target of rapamycin (mTOR) signalling with live-cell imaging and protein analysis, we reveal that cumulus cells not only accelerate maturation but also actively enforce genome quality control through SAC-dependent pathways. Importantly, this sensitization is specific to DNA damage, as cumulus cells do not enhance checkpoint responses to minor spindle defects.

Overall, our study uncovers a previously underappreciated function of cumulus cells in post-GVBD quality control, challenging the prevailing view that denuded oocytes accurately reflect oocyte checkpoint competence. These findings provide new insights into cumulus–oocyte communication and have implications for improving oocyte quality assessment during *in vitro* maturation.

## RESULTS

### Cumulus cells accelerate oocyte meiotic maturation

**Fig. 1.**
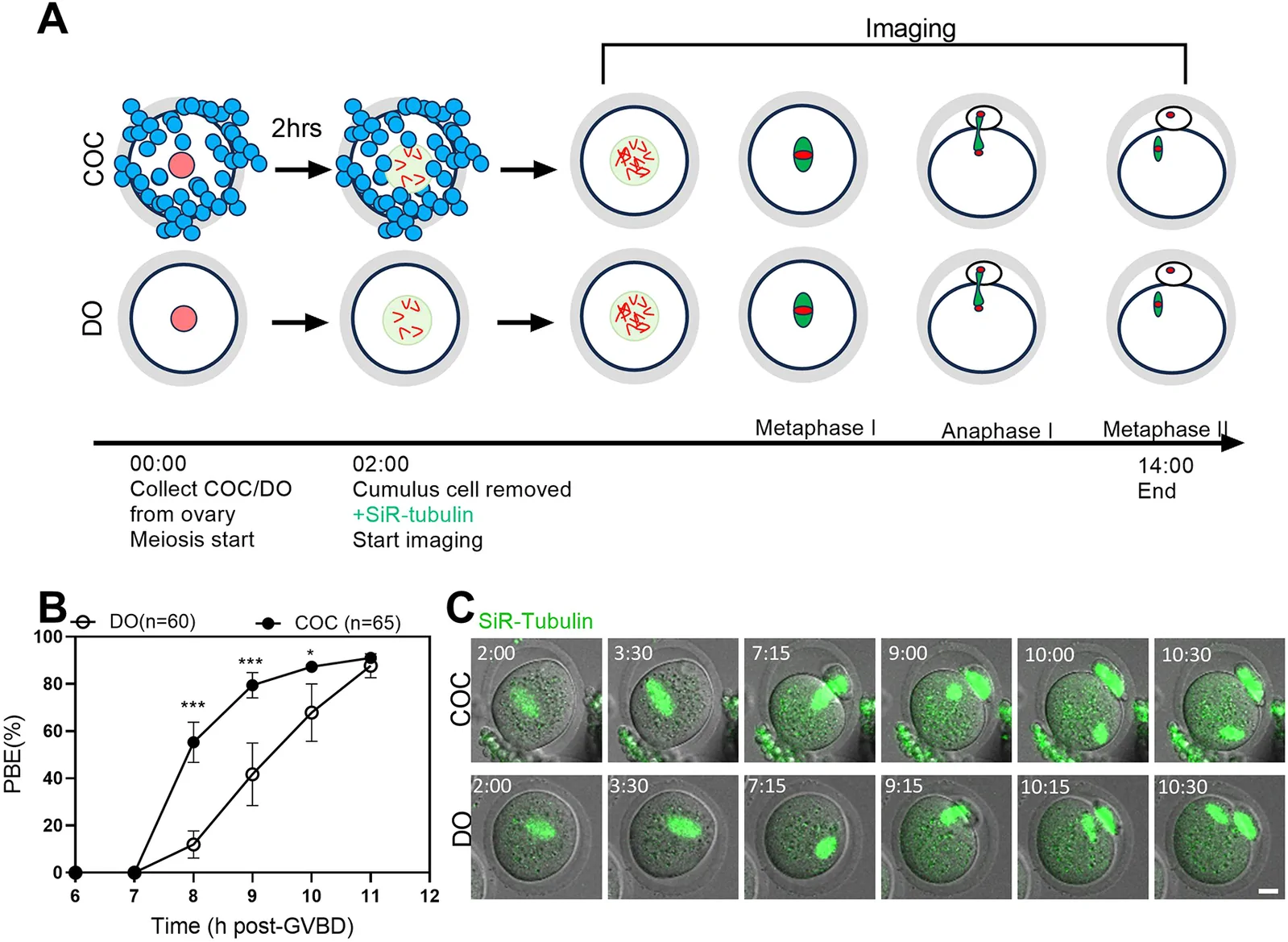
**Meiotic maturation dynamic of oocytes from COCs and DOs.** (A) Schematic illustration of the experimental conditions for meiotic maturation of DOs and COCs. Both groups initiated meiosis immediately after removal from the ovary in M16 medium. Cumulus cells of the COC group were removed after 2 h of incubation, followed by SiR-tubulin loading to label the spindle, and then subjected to time-lapse imaging for 12 h to monitor meiotic maturation. DOs were cultured under the same conditions and imaged by confocal microscopy at 2 h after meiosis and lasting 12 h. Time is indicated as hh:mm post-GV release. (B) Time course of PBE obtained from confocal time-lapse imaging of oocytes originating from DO or COC groups. ***P<0.001; *P<0.05 (two-tailed paired Student's *t*-test). Comparisons were made between DO and COC PBE rates at each time point. *n*=3 independent experiments, with a total of 65 COC oocytes and 60 DO oocytes analysed. (C) Representative time-lapse images showing meiotic maturation of oocytes originating from DOs and COCs. *n*=3 independent experiments, with a total of 65 COC oocytes and 60 DO oocytes analysed. Time is indicated as hh:mm post-GVBD release. Scale bar: 10 μm.

To investigate the role of cumulus cells in oocyte maturation, we compared the dynamics of DOs and COCs. Time-lapse confocal live-cell imaging of SiR-tubulin-labelled spindles was performed over 12 h. To avoid imaging interference from cumulus cells, COCs were denuded after 2 h of incubation (Fig. 1A), ensuring that both groups were cumulus-free during live imaging. Oocytes from COCs underwent first polar body extrusion (PBE) faster than DOs (Fig. 1B,C). Specifically, the PBE rate in COCs reached 55.3±8.5% at 8 h post-GVBD and 79.5±5.4% at 9 h, compared with 12±2.1% and 41.7±13.2% in DOs, respectively (mean±s.e.m.; Fig. 1B,C), indicating an ∼1 h acceleration to reach half-maximal PBE in the COC group. By 11 h post GVBD, PBE rates were similar between groups (91% in COCs versus 87.7% in DOs), demonstrating that cumulus–oocyte communication primarily accelerates meiotic progression without affecting the final maturation rate (Fig. 1B,C).

### Cumulus cells sensitize oocytes to DNA damage-induced meiotic arrest

**Fig. 2.**
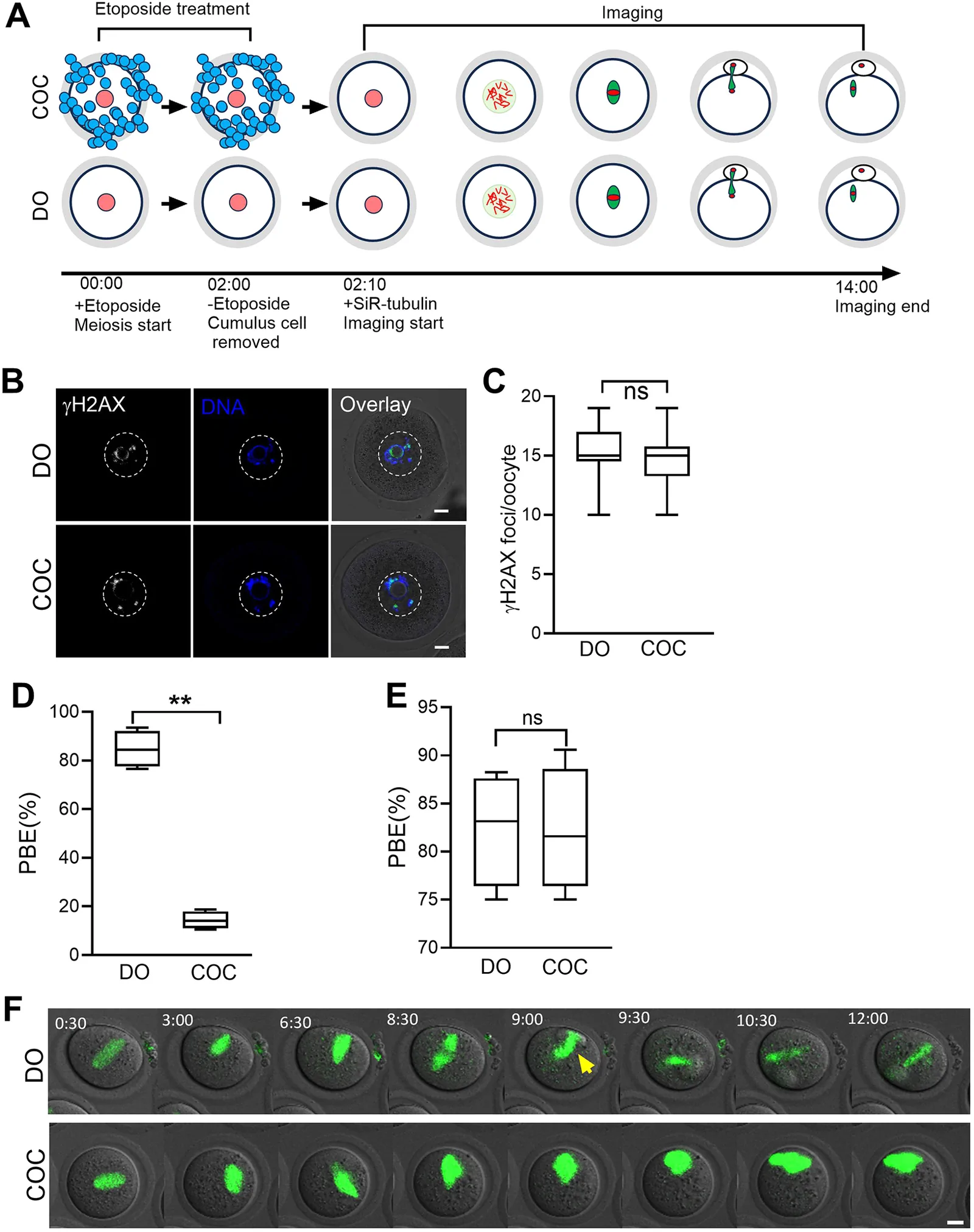
**DNA damage causes metaphase arrest of the oocytes from COCs but not DOs.** (A) Schematic of meiotic maturation in DOs and COCs treated with 10 μg/ml etoposide for 2 h at meiosis onset. Both groups were cultured in M16 medium with etoposide. After 2 h, cumulus cells in COCs group were removed and etoposide were washed out, followed by SiR-tubulin loading and 12 h time-lapse confocal imaging. Time is indicated as hh:mm post-GV release. (B) Representative γH2AX and DNA labelling in DO and COC oocytes. Scale bars: 10 μm. Oocytes were cultured in M16 medium with 10 μg/ml etoposide for 2 h, then fixed and stained with γH2AX antibody and Hoechst. Dashed circles indicate the germinal vesicle location. *n*=4 independent experiments, with a total of 20 COC oocytes and 21 DO oocytes analysed. (C) γH2AX foci per oocyte in COCs and DOs after 2 h etoposide treatment. Data are presented as box-and-whisker plots. *n*=4 independent experiments, with a total of 20 COC oocytes and 21 DO oocytes analysed. ns, not significant (two-tailed paired Student's *t*-test). (D) PBE rates of DO and COC oocytes after 2 h treatment with 10 μg/ml etoposide at maturation onset. Cumulus cells in COCs were removed after 2 h, then cultured in normal M16 for 12 h. Data are presented as box-and-whisker plots. *n*=4 independent experiments, with a total of 91 COC oocytes and 75 DO oocytes analysed. **P<0.01 (two-tailed paired Student's *t*-test). (E) PBE rates of DOs and COCs treated with nocodazole (40 nM). DOs and COCs were cultured in M16 medium for 2 h, after which cumulus cells were removed from the COCs. Oocytes from both groups were then incubated in M16 medium supplemented with nocodazole (40 nM) for an additional 12 h to allow maturation. The percentage of PBE was analyzed at the end of the experiment. Data are presented as box-and-whisker plots. n=4 independent experiments, with a total of 63 COC oocytes and 70 DO oocytes analysed. ns, not significant (two-tailed paired Student's *t*-test). (F) Representative time-lapse images of meiotic maturation in oocytes from DOs and COCs treated with etoposide (10 μg/ml) for 2 h at the beginning of maturation. After 2 h, cumulus cells in the COC group were removed and etoposide was washed out, followed by SiR-tubulin loading and 12 h time-lapse confocal imaging. Arrow indicates anaphase. n=4 independent experiments, with a total of 91 COC oocytes and 75 DO oocytes analysed. Time is shown as hh:mm post-etoposide treatment. Scale bar: 10 μm. For all box-and-whisker plots, the box represents the 25–75th percentiles, and the median is indicated. The whiskers show the full range.

To determine whether cumulus cells contribute to oocyte quality control mechanisms such as DNA damage–induced meiotic arrest, we next examined the response of DOs and COCs to genotoxic stress. Oocytes were treated with etoposide (10 μg/ml, ∼17 μM) for 2 h at the onset of maturation, followed by drug removal and continued culture for 12 h (Fig. 2A). This concentration was selected based on previous studies showing that etoposide at ≤10 μg/ml (≈17 μM) does not induce metaphase I arrest, whereas higher doses (≥25 μg/ml, ≈42 μM) trigger SAC-dependent meiotic block in mouse oocytes (Collins et al., 2015). DNA damage was confirmed by γH2AX staining, which revealed comparable levels of DNA damage in DOs and COCs (Fig. 2B,C). Despite a similar extent of DNA lesions, oocytes from COCs failed to extrude the first polar body and remained arrested at metaphase I, whereas DOs resumed meiosis and reached metaphase II at the normal level (Fig. 2D,F), consistent with previous observations (Collins et al., 2015). These findings indicate that cumulus–oocyte communication sensitizes oocytes to DNA damage-induced meiotic arrest.

We next tested whether cumulus cells influence the response to spindle perturbation. Low-dose nocodazole (40 nM), which causes mild spindle abnormalities, including shortened spindles and chromosome misalignment, without complete spindle disruption (Fig. S1), was applied during maturation. In contrast to the DNA damage response, no significant difference in PBE rate was observed between DOs and COCs under these conditions (Fig. 2E), suggesting that cumulus cells specifically promote checkpoint activation in response to DNA damage, but not for subtle spindle defects.

To determine whether the differential responses of COCs and DOs to DNA damage and minor spindle defects were not a non-specific effect of conventional spontaneous *in vitro* maturation, we examined meiotic progression using a biphasic C-type natriuretic peptide (NPPC)–epidermal growth factor (EGF) culture system, which more closely recapitulates the physiological regulation of meiotic resumption *in vivo*. COCs were maintained in meiotic arrest with 100 nM NPPC and subsequently stimulated to resume meiosis with EGF in the presence of etoposide or low-dose nocodazole (Fig. S2A). DOs were cultured in parallel under the same conditions; however, NPPC is not expected to affect DOs because they lack cumulus cells and, consequently, NPPC receptor signalling. Consistent with observations obtained under conventional maturation conditions, etoposide-treated COCs underwent chromosome condensation and spindle assembly but subsequently arrested at metaphase, whereas DOs progressed through meiosis despite the presence of DNA damage (Fig. S2A,B). In contrast, low-dose nocodazole treatment did not induce metaphase arrest in either COCs or DOs (Fig. S2C). Thus, the distinct response to DNA damage, but not minor spindle perturbation, was preserved under NPPC–EGF culture conditions, demonstrating that this phenotype is independent of the mode of *in vitro* maturation.

### SAC mediates cumulus cell-induced DNA damage sensitization in COCs

**Fig. 3.**
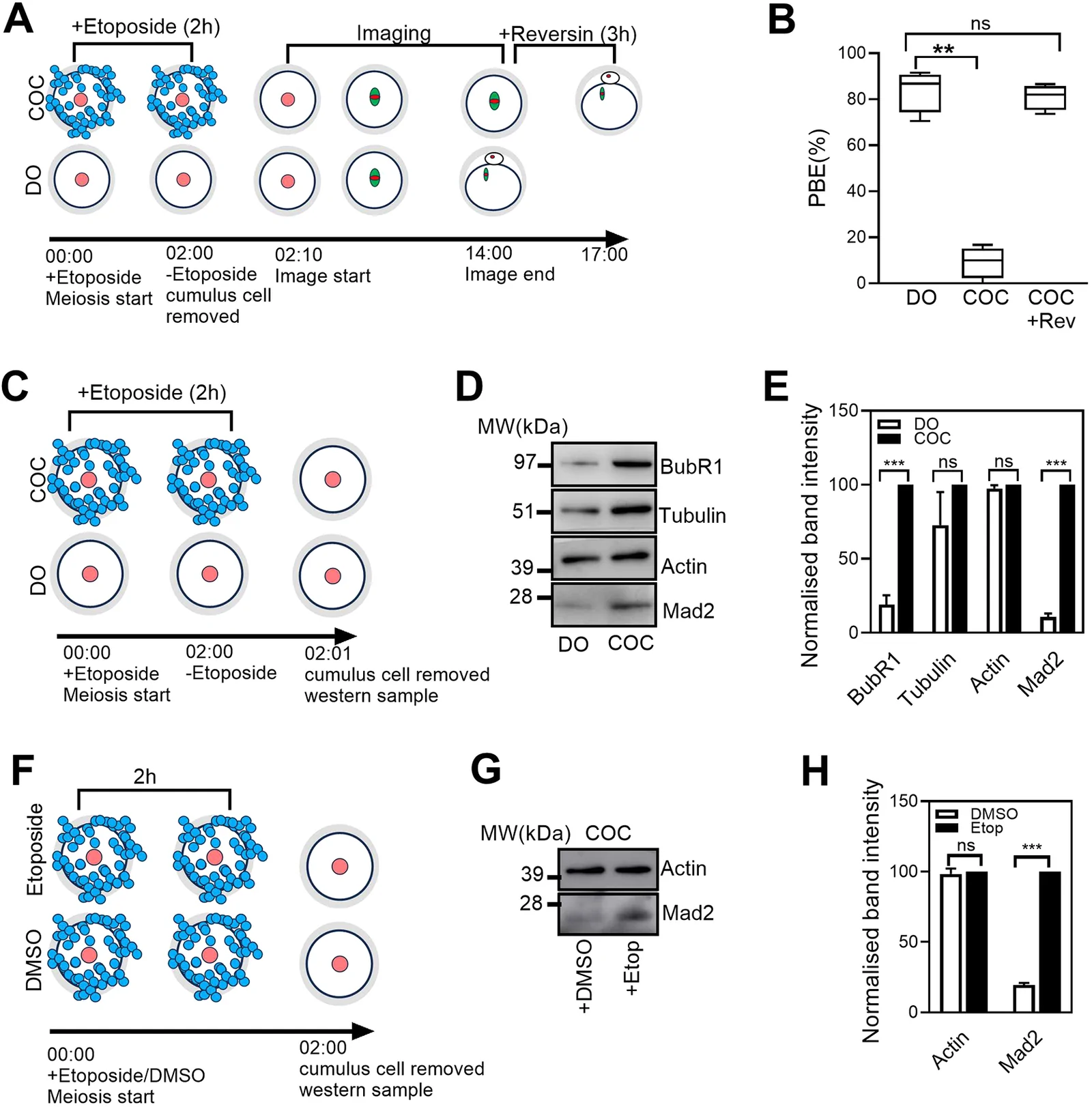
**DNA damage induced meiosis arrest in oocytes from COCs were mediated by SAC.** (A) Schematic illustration of the experimental design showing etoposide treatment of DOs and COCs during meiotic maturation, and the rescue strategy for DNA-damaged, meiosis-arrested oocytes using reversine. Both groups were cultured in M16 medium containing etoposide for the first 2 h of maturation. After 2 h, cumulus cells in the COC group were removed, and etoposide was washed out from both groups, followed by SiR-tubulin loading and 12 h time-lapse confocal imaging. The PBE rate of DOs and COCs was analysed at 14 h. The COC group were then treated with reversin for an additional 3 h to determine the rescue rate of PBE (COC+Rev). Time is indicated as hh:mm post-GV release. (B) Rates of PBE in DNA-damaged oocytes following the experimental conditions described in A. Data are presented as box-and-whisker plots; the box represents the 25–75th percentiles, and the median is indicated. The whiskers show the full range. *n*=4 independent experiments. **P<0.01; ns, not significant (two-tailed paired Student's *t*-test). (C) Schematic illustration of the experimental design showing sample collection for western blot analysis following etoposide treatment of DOs and COCs during meiotic maturation. (D) Oocyte samples (*n*=40) following the experimental conditions described in (C) were immunoblotted for BubR1, tubulin, actin and Mad2. Shown is a representative immunoblot from three independent experiments. (E) Quantification of western blot band intensities normalized to values of COCs. Data are presented as mean±s.e.m. *n*=3 independent experiments. ***P<0.001; ns, not significant (two-tailed paired Student's *t*-test). (F) Schematic illustration of the experimental design showing sample collection following etoposide or DMSO treatment of COCs during meiotic maturation. (G) Representative immunoblot of oocytes (*n*=40) following the experimental conditions described in (F), probed for Actin and Mad2. (H) Quantification of band intensities normalized to COCs treated with etoposide. Data are presented as mean±s.e.m. *n*=3 independent experiments. ***P<0.001; ns, not significant (two-tailed paired Student's *t*-test).

We next investigated the mechanism by which cumulus cells enhance oocyte sensitivity to DNA damage-induced meiotic arrest. The spindle assembly checkpoint has been reported as the primary pathway mediating DNA damage-induced arrest during oocyte maturation (Collins et al., 2015; Lane et al., 2012). To test whether the SAC contributes to the cumulus-dependent arrest in COCs treated with low dose etoposide, we treated DNA-damaged COC oocytes with reversine, an Mps1 kinase inhibitor that suppresses SAC activity (Fig. 3A). Reversine treatment effectively rescued the meiotic arrest in COC oocytes, resulting in a PBE rate of 54.6±9.6% (mean±s.e.m.; COC+reversine, Fig. 3B), which was not significantly different from that of DO oocytes (Fig. 3B, DO, 73.7±10.8%).

To confirm SAC activation, we analysed key SAC components by western blot. BubR1 and Mad2 protein levels were markedly elevated in low-dose etoposide-treated COC oocytes but remained low in DOs (Fig. 3C–E). Moreover, Mad2 expression was significantly higher in etoposide-treated COCs than in DMSO-treated controls (Fig. 3F–H). These results demonstrate that cumulus cell-mediated sensitization of oocytes to DNA damage-induced arrest operates through activation of the SAC pathway.

### Gap junctions and mTOR signalling are essential for DNA-damage induced arrest in COCs

**Fig. 4.**
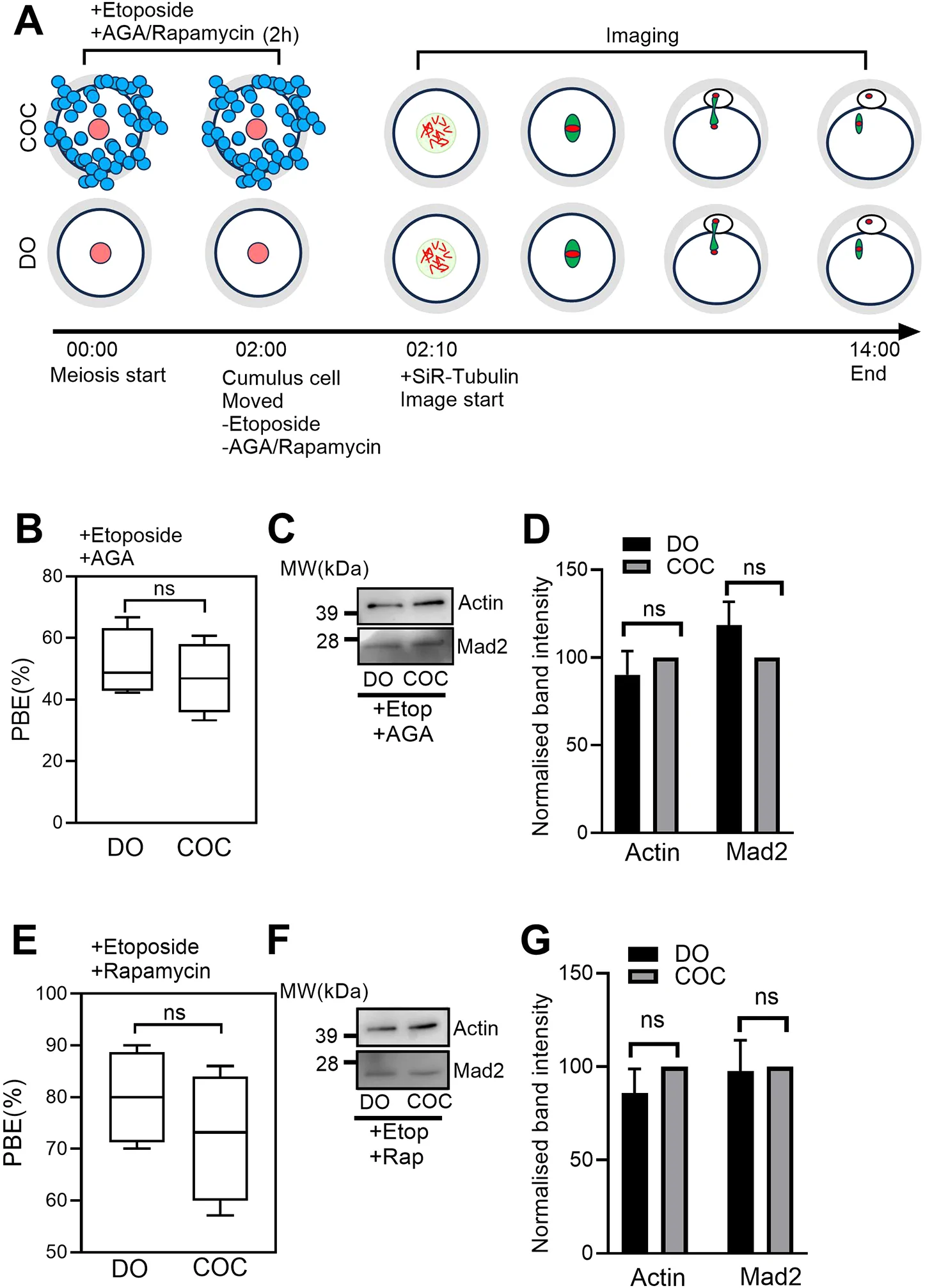
**Effects of gap junction and mTOR pathway inhibition on etoposide-treated oocytes.** (A) Schematic illustration of the experimental design showing etoposide treatment combined with the gap junction inhibitor AGA or the mTOR pathway inhibitor rapamycin in DOs and COCs during meiotic maturation. Both groups were cultured in M16 medium containing etoposide+AGA or etoposide+rapamycin for the first 2 h of maturation. After 2 h, cumulus cells in the COC group were removed, and etoposide, AGA and rapamycin were washed out from both groups, followed by SiR-tubulin loading and 12 h time-lapse confocal imaging. The PBE rate of DOs and COCs was analysed at 14 h. Time is indicated as hh:mm post-GV release. (B) Rates of PBE in DOs and COCs treated with etoposide (10 μg/ml) and AGA for 2 h at the beginning of maturation. Cumulus cells of the COC group were removed after 2 h of incubation, and oocytes were then cultured in normal M16 medium for another 12 h for maturation. Data are presented as box-and-whisker plots. *n*=4 independent experiments, with a total of 63 COC oocytes and 55 DO oocytes analysed. ns, not significant (two-tailed paired Student's *t*-test). (C) Representative immunoblot of actin and Mad2 in oocytes (*n*=40) from DOs and COCs treated with etoposide (10 μg/ml) and AGA for 2 h at the beginning of maturation. Samples were collected immediately after etoposide and AGA treatment. Shown is a representative immunoblot from three independent experiments. (D) Quantification of western blot band intensities normalized to values of COCs. Data are presented as mean±s.e.m. *n*=3 independent experiments. ns, not significant (two-tailed paired Student's *t*-test). (E) Rates of PBE in DOs and COCs treated with etoposide (10 μg/ml) and the mTOR inhibitor rapamycin for 2 h at the beginning of maturation, as described in A. Data are presented as box-and-whisker plots. *n*=4 independent experiments, with a total of 50 COC oocytes and 45 DO oocytes analysed. ns, not significant (two-tailed paired Student's *t*-test). (F) Representative immunoblot of actin and Mad2 in oocytes (*n*=40) from DOs and COCs treated with etoposide (10 μg/ml) and rapamycin for 2 h at the beginning of maturation. Samples were collected immediately after etoposide and rapamycin treatment. Shown is a representative immunoblot from three independent experiments. (G) Quantification of western blot band intensities normalized to values of COCs. Data are presented as mean±s.e.m. *n*=3 independent experiments. ns, not significant (two-tailed paired Student's *t*-test). For all box-and-whisker plots, the box represents the 25–75th percentiles, and the median is indicated. The whiskers show the full range.

To determine how cumulus cells enhance oocyte sensitivity to DNA damage-induced meiotic arrest, we examined the involvement of gap junction communication and mTOR signalling pathways. Cumulus cells are known to couple with oocytes through gap junctions, enabling bidirectional exchange of ions, metabolites and regulatory factors essential for oocyte maturation (Eppig, 2001; Sugiura et al., 2007). To test whether these junctions mediate the cumulus-dependent arrest, we inhibited gap junctions using 18-α-glycyrrhetinic acid (AGA) (Fig. 4A). AGA treatment abolished the difference in PBE rates between DOs and COCs following etoposide exposure, indicating that disruption of gap junction communication prevented the cumulus-induced arrest (Fig. 4B). Consistent with this, western blot analysis revealed that etoposide+AGA treatment did not increase Mad2 protein levels in COCs, and both DOs and COCs exhibited comparable expression (Fig. 4C,D).

Because mTOR signalling in cumulus cells has been implicated in regulating oocyte translation and developmental competence (Chen et al., 2013; Assidi et al., 2008), we next tested whether this pathway contributes to the DNA damage response. COCs and DOs were treated with etoposide in the presence of the mTOR inhibitor rapamycin. Rapamycin similarly abolished the difference in PBE rates between DOs and COCs (Fig. 4E), and no elevation of Mad2 was detected in COCs (Fig. 4F,G). These findings indicate that gap junction-mediated and mTOR-dependent signalling are essential for cumulus cell-induced sensitization of oocytes to DNA damage-triggered meiotic arrest.

## DISCUSSION

Cumulus cells are well known for maintaining oocyte arrest at prophase I, primarily through cAMP-dependent signalling pathways that prevent premature meiotic resumption (Eppig, 2001; Sugiura et al., 2007). Beyond this role, however, their contributions to subsequent stages of oocyte maturation remain poorly understood. It has generally been assumed that, following meiotic resumption, cumulus cells become functionally decoupled from the oocyte and do not significantly influence later maturation events, including spindle assembly and polar body extrusion (Albertini et al., 2001). Our findings challenge this notion, demonstrating that cumulus cells continue to actively regulate oocyte quality control after meiotic resumption by sensitizing oocytes to DNA damage and promoting SAC-dependent metaphase I arrest.

Our results reveal that cumulus cells not only accelerate oocyte meiotic progression but also enhance oocyte sensitivity to DNA damage-induced metaphase I arrest. Time-lapse imaging showed that oocytes from COCs extruded the first polar body earlier than denuded oocytes (DOs), consistent with cumulus-mediated metabolic and paracrine support (Eppig, 2001; Sugiura et al., 2007). This accelerated progression likely reflects more efficient exchange of metabolites and signalling molecules through gap junctions, promoting timely maturation-promoting factor activation.

Despite similar levels of DNA damage, COC oocytes arrested at metaphase I following etoposide or UV exposure (data not shown), whereas DOs resumed meiosis and extruded the first polar body. These findings indicate that cumulus cells sensitize oocytes to checkpoint activation rather than increasing DNA lesions, representing a form of meiotic quality control that prevents propagation of damaged genomes. Because meiotic resumption during conventional *in vitro* maturation occurs spontaneously following follicle isolation, it could be argued that the DNA damage-induced metaphase arrest observed in COCs reflects an artefact of the culture system. To address this possibility, we employed a biphasic NPPC–EGF maturation protocol that preserves the physiological cGMP-dependent signalling axis between cumulus cells and the oocyte, which normally maintains prophase I (GV) arrest within the follicle prior to the LH surge. We observed the same phenotype using a biphasic NPPC–EGF maturation protocol that more closely mimics the hormonal regulation of meiotic resumption *in vivo*. The persistence of metaphase arrest in DNA-damaged COCs, but not DOs, under these conditions suggests that the response is not dependent on spontaneous meiotic entry. Instead, it supports the conclusion that cumulus cells actively mediate a DNA damage-responsive pathway capable of restraining meiotic progression after germinal vesicle breakdown.

Traditionally, mammalian oocytes have been considered to possess a relatively weak SAC compared with somatic cells, as evidenced by frequent aneuploidy and the limited ability of denuded oocytes to arrest in response to spindle or DNA damage (Homer et al., 2005; Rieder and Maiato, 2004). Our findings challenge this notion by demonstrating that cumulus cells confer a highly sensitive DNA damage response. In the presence of cumulus cells, DNA-damaged COCs activate SAC signalling robustly, resulting in metaphase I arrest, whereas the same oocytes, when denuded, fail to mount a checkpoint response. This highlights a previously underappreciated role of cumulus–oocyte communication in maintaining genomic integrity and underscores the limitations of using denuded oocytes as the sole model for oocyte checkpoint studies.

Mechanistically, SAC activation mediates this cumulus-dependent arrest. Reversine treatment rescued PBE in DNA-damaged COCs, and BubR1 and Mad2 protein levels were elevated in damaged COCs but not DOs (Collins et al., 2015; Lane et al., 2012). Disruption of gap junctions with AGA abolished the differential arrest and normalized Mad2 expression, implicating intercellular communication in transmitting damage signals. Similarly, mTOR inhibition with rapamycin prevented cumulus-dependent arrest and Mad2 accumulation, suggesting that mTOR-mediated translational or metabolic regulation in cumulus cells contributes to oocyte checkpoint competence (Chen et al., 2013).

Surprisingly, we did not observe a similar cumulus cell-dependent sensitization in response to spindle perturbation. Low-dose nocodazole, which induces minor spindle defects without fully disrupting spindle assembly, did not affect PBE rates in either DOs or COCs. This indicates that cumulus cells specifically enhance oocyte sensitivity to DNA damage rather than to general spindle defects. This selective checkpoint sensitization is intriguing and suggests that cumulus cells might provide a targeted mechanism for genome quality control. The underlying molecular basis and potential developmental benefits of this specificity warrant further investigation.

Together, these results support a model in which cumulus–oocyte communication integrates maturation acceleration with genome surveillance. Under normal conditions, gap junction- and mTOR-dependent signalling promote timely meiotic progression; upon DNA damage, the same pathways facilitate SAC activation and metaphase I arrest, preventing release of genetically compromised oocytes. These findings expand the known functions of cumulus cells and provide important insights for improving *in vitro* maturation protocols while maintaining oocyte genomic integrity.

### Limitations of the study

We acknowledge that etoposide treatment is expected to induce DNA damage not only in the oocyte but also in the surrounding cumulus cells. Consequently, the metaphase arrest and SAC activation observed in oocytes within COCs might result from a combination of direct DNA damage to the oocyte and indirect effects mediated by damaged cumulus cells. Because etoposide was applied to intact COCs, the present experimental design does not allow us to distinguish between these two possibilities. Unfortunately, our current laboratory setup does not have the ability to induce selective induction of DNA damage in either the oocyte or cumulus cells alone. Addressing this question would likely require specialized approaches, such as targeted delivery of DNA-damaging agents specifically to the oocyte while leaving the surrounding cumulus cells unaffected, or the development of genetic or pharmacological models in which cumulus cells are selectively protected from etoposide-induced DNA damage. To our knowledge, such approaches are not currently established or widely available in the oocyte research field, making it challenging to experimentally separate the direct effects of etoposide on the oocyte from indirect effects mediated by damaged cumulus cells.

Therefore, we cannot exclude the possibility that signals released from DNA-damaged cumulus cells contribute to the observed oocyte response. Although our findings demonstrate that the presence of cumulus cells is associated with enhanced SAC-mediated arrest following DNA damage, further studies using approaches that selectively induce or prevent DNA damage in either the oocyte or cumulus cells will be required to determine whether this response reflects a physiological regulatory mechanism of cumulus–oocyte communication or a pathological consequence of cumulus cell damage.

## MATERIALS AND METHODS

### Drugs and chemicals

Stock solutions of all small-molecule inhibitors were prepared in DMSO at the highest concentration that allowed complete solubilization, minimizing the final DMSO concentration in culture medium (<0.1%).

Etoposide (Sigma-Aldrich, Cat. E1383) was prepared as a 10 mM stock in DMSO and used at a final concentration of 10 μg/ml (≈17 μM) to induce mild DNA damage. 18-α-Glycyrrhetinic acid (AGA) (Sigma-Aldrich, Cat. G8503) was prepared as a 10 mM stock in DMSO and used at a final concentration of 5 μM to inhibit gap-junction communication. Reversine (Sigma-Aldrich, Cat. R3904) was prepared as a 10 mM stock in DMSO and used at a final concentration of 0.5 μM to inhibit spindle-assembly checkpoint signalling. Rapamycin (Sigma-Aldrich, Cat. R8781) was prepared as a 10 mM stock in DMSO and used at a final concentration of 100 nM to inhibit mTOR signalling. Nocodazole (Sigma-Aldrich, Cat. M1404) was prepared as a 10 mM stock in DMSO and used at a final concentration of 40 nM to induce mild spindle perturbation. SiR-tubulin (Spirochrome, Cat. SC002) was 1 mM stock in DMSO used at a final concentration of 100 nM for live-cell spindle imaging according to the manufacturer's protocol. NPPC (Sigma-Aldrich, Cat. No. N8768) was prepared as a 100 μM stock solution in PBS containing 0.1% BSA and used at a final concentration of 100 nM to maintain meiotic arrest in cumulus–oocyte complexes. EGF (Sigma-Aldrich, Cat. No. E9644) was prepared as a 10 μg/ml stock solution in PBS containing 0.1% BSA and used at a final concentration of 10 ng/ml to promote cumulus cell expansion and support meiotic resumption. All working solutions were freshly prepared on the day of the experiment.

### Collection and culture of COCs and DOs

All animals were housed in a specific pathogen-free environment in filter-top cages and fed a standard diet. All animal procedures were conducted in accordance with institutional guidelines and approved by the Animal Ethics Committee of the University of Queensland.

Female B6CBAF1 mice (3–4 weeks old) were superovulated by intraperitoneal injection of 7.5 IU pregnant mare's serum gonadotrophin (PMSG; Pacific Vet). Ovaries were collected 44–46 h post injection and transferred to pre-warmed M2 medium. Antral follicles were punctured with a 27-gauge needle under a stereomicroscope (M165C, Leica Microsystems) to release oocytes. Fully grown COCs were selected for subsequent culture.

For DOs, cumulus cells were mechanically removed from COCs by gentle pipetting through a narrow-bore glass capillary. For time-lapse imaging experiments, COCs were initially cultured intact for 2 h to allow meiotic resumption and communication between cumulus cells and oocytes, after which cumulus cells were removed to prevent imaging interference. Both groups were thus imaged under cumulus-free conditions.

Oocytes were cultured in microdrops of M16 medium (Sigma-Aldrich) under mineral oil (Sigma-Aldrich) at 37°C in a humidified atmosphere of 5% CO_2_ in air. Oocytes were maintained at the GV stage or allowed to mature *in vitro* according to the experimental design.

### *In vitro* meiotic maturation of COCs under NPPC–EGF culture conditions

COCs (and DOs where indicated) were cultured in α-MEM (Gibco, cat. no. 12000-063) supplemented with 3 mg/ml BSA (Merck, cat. no. A8806) and 100 nM NPPC at 37°C in a humidified atmosphere of 5% CO_2_ for 1 h to maintain GV arrest. COCs or DOs were then washed three times in maturation medium to remove residual NPPC and transferred to α-MEM supplemented with 5% fetal bovine serum (FBS; Gibco, cat. no. A5209401) and 10 ng/ml EGF. Oocytes were cultured under the same conditions for a further 2 h to initiate meiotic resumption. Cumulus cells were then removed from COCs by gentle pipetting, and oocytes were transferred to imaging medium for live-cell imaging for up to 12 h to monitor chromosome and spindle dynamics during meiotic progression. For experiments involving DNA damage induction, etoposide was added at the beginning of the NPPC culture phase and maintained throughout both the NPPC-mediated arrest phase and the subsequent 2 h EGF-induced maturation phase. A schematic overview of the experimental timeline and treatment regimen is provided in Fig. S2A.

### Immunofluorescence

GV-stage oocytes were fixed as described previously (Gui and Homer, 2012, 2013; Homer et al., 2009; Zhou et al., 2019). Briefly, oocytes were washed in PIPES, HEPES, EGTA and MgCl_2_ (PHEM) buffer (pH 7.0) and prepermeabilized in 0.25% Triton-X in PHEM. Oocytes were then fixed in 3.7% PFA solution in PHEM for 20 min. Oocytes were blocked overnight in 3% BSA in PBS containing 0.05% Tween-20 at 4°C. Primary antibody incubation with anti-γH2AX antibody (Abcam, ab11174; 1:200) was performed for 1 h at 37°C. Following three 5-min washes in PBS containing 0.5% BSA and 0.05% Tween-20, oocytes were incubated with the Alexa Fluor 488-conjugated secondary antibodies (1:200; Thermo Fisher Scientific) for 1 h at 37°C. Oocytes were then washed three times before staining for DNA by incubating in Hoechst 33342 (10 μg/ml; Sigma-Aldrich) for 5 min.

### Immunoblotting

Western blotting was performed as described previously (Zhou et al., 2019, 2022; Wei et al., 2020). Protein samples were prepared by washing oocytes three times in ultra-pure water and lysing directly in LDS sample buffer (NuPAGE, Invitrogen) supplemented with a reducing agent (NuPAGE, Invitrogen). Samples were vortexed, snap-frozen by dry ice and stored at −80°C until use. Before electrophoresis, samples were boiled for 5 min at 95°C. Proteins were separated on 4–12% Bis-Tris gels (NuPAGE, Invitrogen) and transferred to PVDF membranes (Immobilon-P, Millipore).

Membranes were blocked for 1 h at room temperature in 3% BSA in TBST (25 mM Tris-HCl, 150 mM NaCl, 0.05% Tween-20) and incubated overnight at 4°C with primary antibodies diluted in blocking buffer. The following antibodies were used: anti-BubR1 (1:2000; Abcam, ab28193), anti-Mad2 (1:1000; Abcam, ab10691), anti-actin (1:2000; Sigma-Aldrich, A1978), and anti-α-tubulin (1:2000; Sigma-Aldrich, T6199). After three 5-min washes in TBST, membranes were incubated with HRP-conjugated goat anti-rabbit-IgG or goat anti-mouse-IgG secondary antibodies (1:1000; Bio-Rad) for 1 h at room temperature. Protein bands were visualized using Western Lightning ECL-Pro (Perkin Elmer) and imaged on an ImageQuant LAS 500 system (GE Healthcare).

Band intensities were quantified using ImageJ software and normalized to levels of COC group for comparison across samples. Full the western blot data is shown in Fig. S3.

### Confocal microscopy

Imaging was performed using a Leica TCS SP8 confocal microscope equipped with a 20×/0.75 NA Apochromat water-immersion objective fitted with an automated pump cap (water immersion micro dispenser, Leica; automated pump mp-x controller, Bartels Mikrotechnik) as described previously (Zhou et al., 2019; Zhou and Homer, 2022, 2025; Wei et al., 2020).

Time-lapse microscopy was used to simultaneously image spindles and chromosomes (Zhou et al., 2019; Zhou and Homer, 2022, 2025; Wei et al., 2020; Zhou et al., 2025). To visualise microtubules, silicon rhodamine (SiR)-Tubulin dye (Cytoskeleton, Inc.) was added to media at a final concentration of 100 nM as validated previously in mouse oocytes (Zhou et al., 2019, 2022; Zhou and Homer, 2025; Wei et al., 2020). Oocytes were imaged in 5 μl micro-drops of M16 medium in glass-bottom dishes (35×10 mm dish, MatTek) under mineral oil enclosed within a purpose-built stage-mounted incubation chamber designed to maintain conditions of 37°C and 5% CO_2_ in air. Temperature fluctuation was further buffered by enclosing the entire microscope, including the stage-mounted chamber, in a custom-designed polycarbonate incubator (Life Imaging Services) that maintained a stable internal temperature of 37°C. Automated image capture was driven by the Leica LAS X software. At the commencement of imaging, the positions of the oocytes in the *z*-axis were located. The complete stack was then derived by setting a stack thickness of 50 μm. *Z*-stacks were acquired with step intervals of 4 μm at 5 min, and a speed of 600 Hz. Using the Leica mark-and-find tool, we typically imaged multiple groups of oocytes in separate droplets in each experiment. The 633 nm (for SiR-Tubulin) laser lines were used at 3% intensity.

Post-acquisition image processing was performed using Leica LAS X software and images were assembled into panels using Adobe Illustrator (Adobe Systems). Fluorescence images were produced by *Z*-projecting the entire stack of fluorescence images and merging them with images in the bright-field channel.

### Graphical representation and statistical analysis

GraphPad Prism (GraphPad, USA) was used to calculate mean and s.e.m. Data are presented as box-and-whisker plots, where the box represents the interquartile range and the line indicates the median. Whiskers represent the minimum to maximum values. Statistical comparisons were made using a two-tailed paired Student's *t*-test with Welch's correction. Graphs were prepared in GraphPad Prism. *P*-values are represented as *P<0.05, **P<0.01, ***P<0.001, ns (not significant) is *P*>0.05. All experiments were repeated a minimum of three times.

## Supplementary Material

10.1242/joces.264929_sup1 Supplementary information
