# Supplementary material for: Cumulus cells enhance oocyte genomic quality control by promoting DNA damage-induced meiotic arrest
Source: J Cell Sci. 2026 Jul 27;139(14):jcs264929. doi: 10.1242/jcs.264929 (PMC13490734; doi:10.1242/jcs.264929)
Supplement: Supplementary file 1 [file joces-139-264929-s1.pdf]

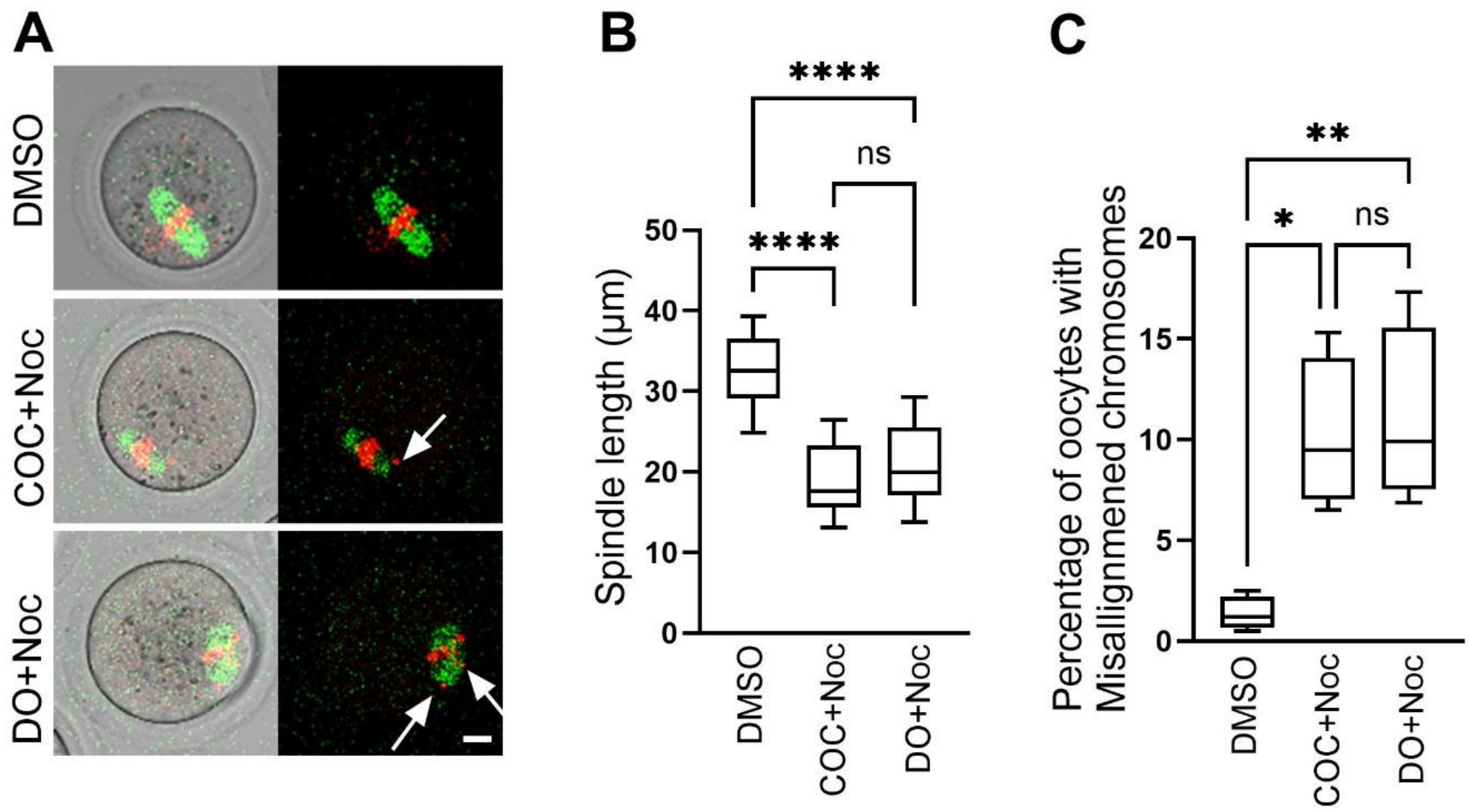

Fig. S1. Low-dose Nocodazole treatment induces minor spindle defects in mouse oocytes.

(A) Representative images of oocytes treated with 0.1% DMSO (control) or 40 nM nocodazole. Chromosomes and spindle microtubules were labeled with SiR-DNA and SPY555-Tubulin, respectively. Arrows indicate misaligned chromosomes. Scale bar = 10 µm.

(B) Quantification of spindle length in oocytes treated with DMSO or 40 nM nocodazole.

(C) Percentage of oocytes exhibiting chromosome misalignment following treatment with DMSO or 40 nM nocodazole.

COC+Noc: COC treated with Nocodazole; DO+Noc: DO treated with Nocodazole. Data are presented as box-and-whisker plots. Each biological replicate represents an independent experiment (n = 4). Statistical analysis was performed using one-way ANOVA. *P < 0.05, **P < 0.01, ****P < 0.0001, ns, not significant (P > 0.05).

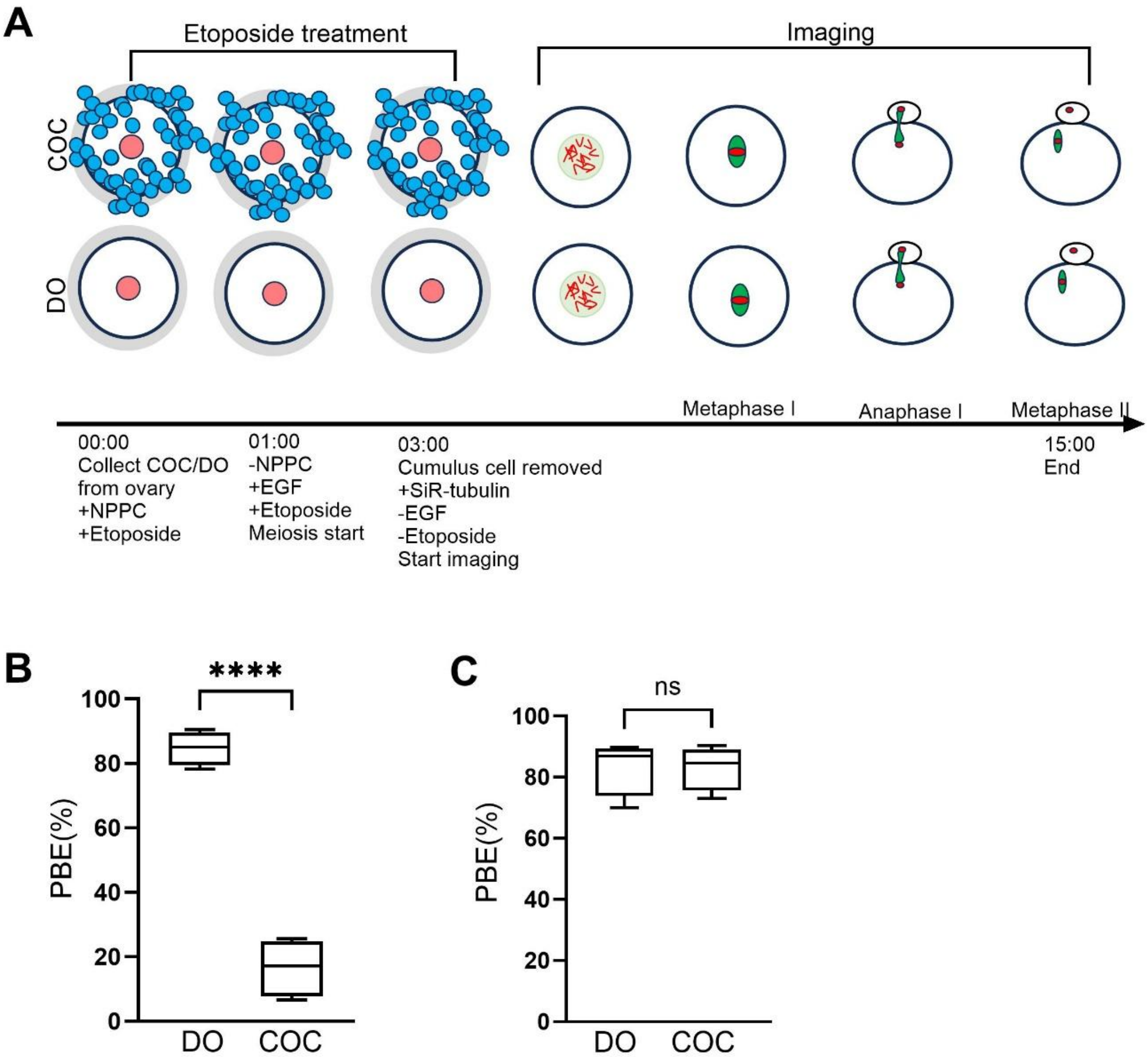

Fig. S2. DNA damage, but not minor spindle defects, induces metaphase arrest in oocytes from COCs but not DOs under NPPC–EGF culture conditions.

(A) Schematic of meiotic maturation in DOs and COCs treated with 10 µg/mL etoposide for 3 h under NPPC–EGF maturation conditions.

(B) Polar body extrusion rates of DOs and COCs following treatment with 10 µg/mL etoposide for 3 h under NPPC–EGF maturation conditions. Data are presented as box-and-whisker plots. n = 3 independent experiments, with a total of 98 DOs and 101 COCs analysed. Statistical significance was determined using Student's t-test. ****$P < 0.0001$.

(C) Polar body extrusion rates of DOs and COCs treated with 40 nM nocodazole under NPPC–EGF maturation conditions. Data are presented as box-and-whisker plots. n = 3 independent experiments, with a total of 90 DOs and 83 COCs analysed. Statistical significance was determined using Student's t-test. ns, not significant ($P > 0.05$).

Journal of Cell Science • Supplementary information

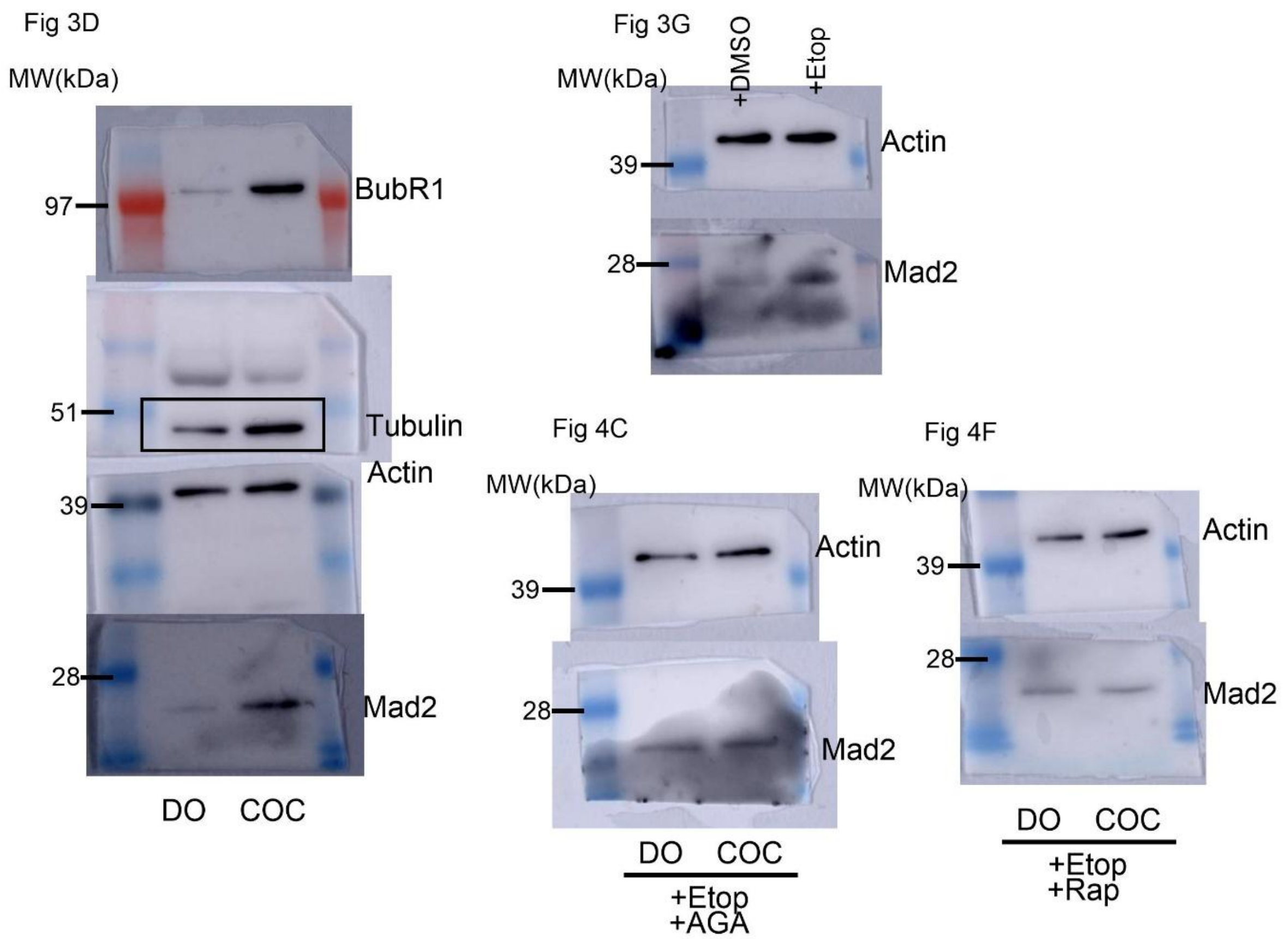

Fig. S3. Original western blots. Black boxes indicate areas of blots featured in text**.**

Journal of Cell Science • Supplementary information
